# The Tick-Derived Anticoagulant Madanin Is Processed by Thrombin and Factor Xa

**DOI:** 10.1371/journal.pone.0071866

**Published:** 2013-08-12

**Authors:** Ana C. Figueiredo, Daniele de Sanctis, Pedro José Barbosa Pereira

**Affiliations:** 1 IBMC – Instituto de Biologia Molecular e Celular, Universidade do Porto, Porto, Portugal; 2 Structural Biology Group, European Synchrotron Radiation Facility (ESRF), Grenoble, France; University of Graz, Austria

## Abstract

The cysteine-less peptidic anticoagulants madanin-1 and madanin-2 from the bush tick *Haemaphysalis longicornis* are the founding members of the MEROPS inhibitor family I53. It has been previously suggested that madanins exert their functional activity by competing with physiological substrates for binding to the positively charged exosite I (fibrinogen-binding exosite) of α-thrombin. We hereby demonstrate that competitive inhibition of α-thrombin by madanin-1 or madanin-2 involves binding to the enzyme's active site. Moreover, the blood coagulation factors IIa and Xa are shown to hydrolyze both inhibitors at different, although partially overlapping cleavage sites. Finally, the three-dimensional structure of the complex formed between human α-thrombin and a proteolytic fragment of madanin-1, determined by X-ray crystallography, elucidates the molecular details of madanin-1 recognition and processing by the proteinase. Taken together, the current findings establish the mechanism of action of madanins, natural anticoagulants that behave as cleavable competitive inhibitors of thrombin.

## Introduction

Ticks are parasitic arthropods that feed on mammalian blood, a task that is assisted by the action of a range of antihemostatic compounds present in their saliva, eggs and hemolymph [1]. In addition to preventing host blood coagulation, these compounds have been postulated to play a role in tick hemolymph coagulation itself [2].

The bush tick *Haemaphysalis longicornis* belongs to the *Ixodidae* or hard-tick family. This family represents 80% of the world's tick fauna and its global economic importance is mostly related to its impact on livestock [3]. Adult female hard ticks feed only once (dying after oviposition) but for a prolonged period of time [4]. This feeding habit allows for both delivery and uptake of blood-borne parasites, thus explaining the role of ticks as important vectors of livestock-affecting diseases [5]. The genus *Haemaphysalis* is mostly prevalent in tropical areas and is characterized by small size inortate ticks with short mouthparts (brevirostrate) [6]. Of particular concern is the impact of *H. longicornis* in the livestock industry in Japan and other East Asian countries, mainly due to the transmission of the anemia-causing intraerythrocytic protozoa, *Theileria sergenti* and *Th. buffeli*.

Two isoforms of a thrombin inhibitor, madanin-1 and madanin-2, sharing 79% amino acid sequence identity, were identified in salivary gland cDNA libraries of *H. longicornis* ticks [7], [8]. Given the lack of sequence similarity to other inhibitors, madanins were classified as a distinct family - family I53 - of the MEROPS database [9]. Madanins display two clusters of acidic residues in the N-terminal two thirds of their amino acid sequence, conferring on them an overall acidic character (theoretical pI of 4.84 and 4.50 for madanin-1 and madanin-2, respectively). Another distinctive feature of madanins is the absence of cysteine residues in their amino acid sequence, placing them in the restricted group of cysteine-less thrombin inhibitors, together with thrombostasin (from the horn fly *Haematobia irritans* [10]; MEROPS family I64), tsetse thrombin inhibitor (from *Glossina morsitans morsitans* [11]; MEROPS family I76), chimadanin (from *H. longicornis* [12]; MEROPS family I72), anophelin (from *Anopheles* mosquitoes [13]; MEROPS family I77) and variegin (from the tropical bont tick *Amblyomma variegatum* [14]; MEROPS family I74). The molecular mechanism of action of two of these atypical serine proteinase inhibitors, anophelin and variegin, has been unveiled by the crystallographic three-dimensional structures of their complexes with thrombin [15], [16]. Whereas anophelin and variegin bind tightly to thrombin, madanins were shown to bind to thrombin with low affinity [7]. Further, while both variegin and anophelin occupy the active site of the proteinase, albeit with distinct binding modes and mechanisms of inhibition, madanins were proposed to bind only to thrombin's exosite I [7], [17].

Here we show that inhibitors of MEROPS family I53 are hydrolysed by the blood coagulation factors α-thrombin and factor Xa. In contrast to the also thrombin-cleavable variegin [16], the inhibitory activity of madanins is lost after the cleavage reaction takes place. In addition to prolonging thrombin time (TT) in a dose-dependent manner, and in contrast to previous reports [7], we show that madanins inhibit the amidolytic activity of α-thrombin against a chromogenic substrate and are able to bind to the enzyme's active site. Finally, the crystallographic three-dimensional structure of the complex between human α-thrombin and a madanin-1 fragment elucidated the molecular details of madanin-1 recognition and processing by the proteinase.

## Materials and Methods

### Production of recombinant madanin-1 and madanin-2

Synthetic genes coding for mature *Haemaphysalis longicornis* madanin-1 and madanin-2, with codon usage optimized for expression in *Escherichia coli*, were obtained from GenScript. Madanin-1 and madanin-2 ORFs were subcloned into the SapI and PstI restriction sites of the expression vector pTYB11 (New England BioLabs) in fusion with an N-terminal intein tag. *E. coli* ER2566 cells (New England BioLabs) transformed with pTYB11-madanin-1 or pTYB11-madanin-2 plasmids were grown at 37 °C in lysogeny broth [18] supplemented with 50 µg/ml ampicillin to OD_600_ 0.5, and expression was induced by addition of isopropyl-β-D-thiogalactopyranoside (0.4 mM final concentration). After overnight growth at 18 °C, cells were lysed by sonication in 20 mM Tris-HCl pH 8.5, 500 mM NaCl, 1 mM ethylenediaminetetraacetic acid (buffer A). Clarified protein extracts were loaded onto chitin-agarose columns (New England BioLabs) pre-equilibrated with buffer A, and eluted with buffer A supplemented with 50 mM 1,4-dithiothreitol. Protein-containing fractions were concentrated and further purified on a HiPrep 16/60 Sephacryl S-100 column (GE Healthcare) pre-equilibrated with 20 mM Tris-HCl pH 8.0, 150 mM NaCl.

### Circular dichroism (CD) spectroscopy

Far-UV region (190–260 nm) spectra were recorded in a 1.0 mm path-length quartz cuvette at 20 °C with a Peltier temperature-controlled cell holder-equipped Jasco J-815 spectropolarimeter from a 50 µg/ml protein solution in 20 mM sodium phosphate buffer pH 8.0. Secondary structure content was estimated using the DichroWeb server [19].

### Thrombin time (TT) assays

Human plasma (800 µl) was mixed with 200 µl of recombinant madanin-1 or madanin-2 solution (0, 5 or 10 µM final concentration in 10 mM HEPES pH 7.5, 10 mM NaCl) and thrombin time was measured for each sample at BM Análises Clínicas (http://www.bmac.pt/) using standard protocols.

### Thrombin inhibition assays

The inhibition of the amidolytic activity of titrated [20] bovine α-thrombin (GE Healthcare) was followed spectrophotometrically using Tos-Gly-Pro-Arg-p-nitroanilide (Roche) as chromogenic substrate. Assays were performed using 1 nM thrombin and increasing concentrations (0–200 µM) of substrate in the presence of recombinant proteins (0–800 nM). Reactions were carried out at 37 °C in 50 mM Tris-HCl pH 8.0, 50 mM NaCl, 1 mg/ml bovine serum albumin, and monitored at 405 nm for 1 hour on a Synergy2 multi-mode microplate reader (Biotek). The reactions were started by addition of enzyme. The inhibition constant, *K*_i_, was determined by fitting the data to the competitive inhibition model (GraphPad Prism 5), with R^2^ parameters of 0.989 (madanin-1) and 0.992 (madanin-2). For each inhibitor, at least three independent experiments with duplicate reactions were performed, together with control reactions in the absence of enzyme.

Madanin fragments were also assayed for their ability to inhibit thrombin. Assays were performed using 1 nM thrombin, 50 µM of chromogenic substrate and 1 µM of fragments or full-length proteins (purified by reverse-phase chromatography as described below). For each inhibitor, triplicate reactions were performed, together with control reactions in the absence of enzyme. Inhibition of thrombin was calculated as percentage of inhibition after 120-min reaction.

### Electrophoretic mobility shift assays

Thrombin·madanin complex formation in solution was assessed using non-denaturing PAGE: 30 pmol of human α-thrombin (Haematologic Technologies) were mixed with madanin-1 or madanin-2 (1∶1, 1∶2, 1∶4, 1∶8 molar ratios) and incubated at room temperature for 15 min. Samples were separated at 4 °C in 10% (w/v) polyacrylamide gels (24 mM Tris and 192 mM glycine running buffer) and stained with PageBlue (Fermentas). Proteolytic cleavage of madanin-1 or madanin-2 by α-thrombin was assessed using the same methodology: samples containing 300 pmol madanin-1 or madanin-2, and varying amounts of α-thrombin (0–30 pmol) were incubated at 37 °C for 30 min, separated at 4 °C in 10% (w/v) polyacrylamide gels (24 mM Tris and 192 mM glycine running buffer), and stained with PageBlue (Fermentas).

### Size exclusion chromatography

Three mg of human α-thrombin (Haematologic Technologies) were mixed with a 10% molar excess of purified madanin-1 and incubated on ice for 1 h. The complex was separated from isolated components by size exclusion chromatography on a Superdex 75 HR10/30 column (GE Healthcare) equilibrated in 50 mM HEPES pH 7.4, 125 mM NaCl. For comparison purposes, thrombin (3 mg) and recombinant madanin-1 (6 mg) were individually applied to the same column.

### Cleavage of madanins by thrombin or factor Xa

Madanin-1 or madanin-2 was incubated with human α-thrombin (Haematologic Technologies) or bovine factor Xa (Roche) at a 10∶1 molar ratio. The reactions were quenched after 0 or 2 h incubation at 37°C by addition of 0.1% (v/v) trifluoroacetic acid (TFA, Sigma) before separation by reverse-phase chromatography.

### Reverse-phase chromatography

All reverse-phase chromatography separations were performed at 20 °C using a binary solvent system consisting of 0.1% (v/v) TFA in water (solvent A) and 0.09% (v/v) TFA in gradient-grade acetonitrile (Merck; solvent B). Full-length madanin-1 and its cleavage products were separated on a BioSil 304-10 column (C4-alkyl chains; Bio-Rad) with a linear gradient of 5–55% solvent B in 30 column-volumes and a flow rate of 2 ml/min. Full-length madanin-2 and its cleavage products were separated on a Jupiter 300A C18 column (C18-alkyl chains; Phenomenex) with a linear gradient of 5–45% solvent B in 30 column-volumes and a flow rate of 1 ml/min.

Madanin fragment-containing fractions were pooled and lyophilized. Samples used for TT and thrombin inhibition assays were dissolved in 10 mM HEPES pH 7.5, 10 mM NaCl. Samples used for mass spectrometry (MS) and N-terminal sequencing analysis were dissolved in 20% (v/v) acetonitrile, 0.1% (v/v) TFA.

### Protein sequencing and MS analysis

The N-terminal sequence of full-length madanins and madanin fragments were determined by Edman degradation on a Procise 492 protein sequencer (Applied Biosystems). The molecular mass of the peptides was determined by MALDI-TOF on a 4800 Proteomics Analyser (AB Sciex). Samples were crystallized with α-cyano-4-hydroxycinnamic acid as matrix and analyzed in reflectron mode.

### Crystallization of the thrombin·madanin-1 complex

Thrombin·madanin-1 complex was prepared by mixing human α-thrombin (Haematologic Technologies) with a five-fold molar excess of purified recombinant madanin-1. The complex was concentrated to 19.1 mg/ml on a centrifugal concentration device with a 3 kDa molecular-weight cut-off membrane (Millipore). Crystallization experiments were performed at the EMBL HTX Lab (Grenoble, France) in a Cartesian PixSys 4200 robot (Genomic Solution) using the sitting-drop vapor-diffusion method with commercial sparse-matrix crystallization screens. The experiments were set up in CrystalQuick plates (Greiner) using drops composed of identical volumes (0.1 µl) of protein and precipitant solution, equilibrated against a 88 µl reservoir, and incubated at 20 °C. Crystals were obtained after 7 days using 0.2 M lithium sulfate, 0.1 M Tris-HCl pH 8.5, 25% (w/v) PEG 3350 as precipitant.

### Data collection and processing

Crystals were harvested from the CrystalQuick plates, cryoprotected in mother liquor supplemented with 35% PEG 3350, and flash-cooled in liquid nitrogen. Diffraction data from a single cryo-cooled crystal (100 K) were collected at beamline ID29 [21] of the ESRF (Grenoble) on a Pilatus 6 M detector (Dectris) using a wavelength of 0.9763 Å. One thousand frames were measured in 0.2 ° oscillation steps with a 611.6 mm sample-to-detector distance and 0.125 seconds exposure per frame. Diffraction data were integrated with XDS [22], scaled with XSCALE [23] and reduced with utilities from the CCP4 program suite [24]. Data collection and refinement statistics are summarized in Table 1.

**Table 1:** Data collection and refinement statistics.

**Data collection^a^**	
Space group	P2_1_2_1_2_1_
Unit cell dimensions	a = 53.1; b = 78.6; c = 155.2
Resolution range (Å)	44.0–2.60 (2.74–2.60)
Reflections (measured/unique)	131831/20364
Completeness (%)	97.7 (86.5)
Multiplicity	6.5 (4.1)
R_sym_	0.126 (0.590)
R_pim_	0.052 (0.310)
Mean [(I)/σ (I)]	12.6 (1.9)
Wilson B-factor	46.9
Matthews coefficient (Å^3^ Da^−1^)	2.18
Solvent content (%)	44.0
**Structure refinement^a^**	
Resolution range (Å)	44.0–2.60 (2.73–2.60)
Rfactor^b^/Free Rfactor^c^	0.191/0.239
N° of unique reflections (working/test set)	20308/1035
Water molecules	92
Ions (Na^+^/SO_4_^2−^)	2/4
Total number of atoms	4765
Average overall B-factor (Å^2^)	53.3
Average protein B-factor (Å^2^)	53.1
Average main chain B-factor (Å^2^)	50.6
Average side chain B-factor (Å^2^)	55.6
Average water B-factor (Å^2^)	40.9
r.m.s.d bonded Bs (Å^2^)	2.34
r.m.s.d bond lengths (Å)	0.004
r.m.s.d bond angles (°)	0.855
**Ramachandran plot statistics**	
Favored (%)	95.0
Allowed (%)	4.7
Disallowed (%)	0.4

(a) Values in parenthesis correspond to the outermost resolution shell. The data were recorded from a single crystal.

(b) R_factor_ = ∑||F_o_|−|F_c_||/∑|F_o_| where |F_o_| and |F_c_| are observed and calculated structure factor amplitudes, respectively.

(c) Free R_factor_ is the cross-validation R-factor computed for a randomly chosen subset of 5% of the total number of reflections, which were not used during refinement.

### Structure determination and refinement

The structure of the human α-thrombin·madanin-1 complex was solved by molecular replacement with Phaser [25] using the coordinates of free human α-thrombin as search model (PDB entry 3U69 [26], [27]). Alternating cycles of model building with Coot [28] and of refinement with PHENIX [29]_ENREF_45 were performed until model completion. For one thrombin molecule the final model comprises residues ^T^Gly1F to ^T^Asp14L (chain L, light chain) and ^T^Ile16 to ^T^Gly149D and ^T^Gly150 to ^T^Glu247 (chain H, heavy chain). For the other thrombin molecule the final model comprises residues ^T^Ala1B to ^T^Asp14L (chain B, light chain) and ^T^Ile16 to ^T^Glu146 and ^T^Gly150 to ^T^Phe245 (chain A, heavy chain). A fragment of madanin-1 comprising ^M^Ala51 to ^M^Arg54 (Ala-Lys-Pro-Arg) was modeled (chain M) in complex with one of the protease molecules present in the asymmetric unit (chains L and H). Each of the two molecules of thrombin contains an N-acetyl-glucosamine sugar moiety attached to ^T^N60G in the heavy chain. The final refined coordinates and structure factors were deposited at the Protein Data Bank under accession number 4BOH.

## Results and Discussion

### Production of recombinant madanins

**Figure 1:**
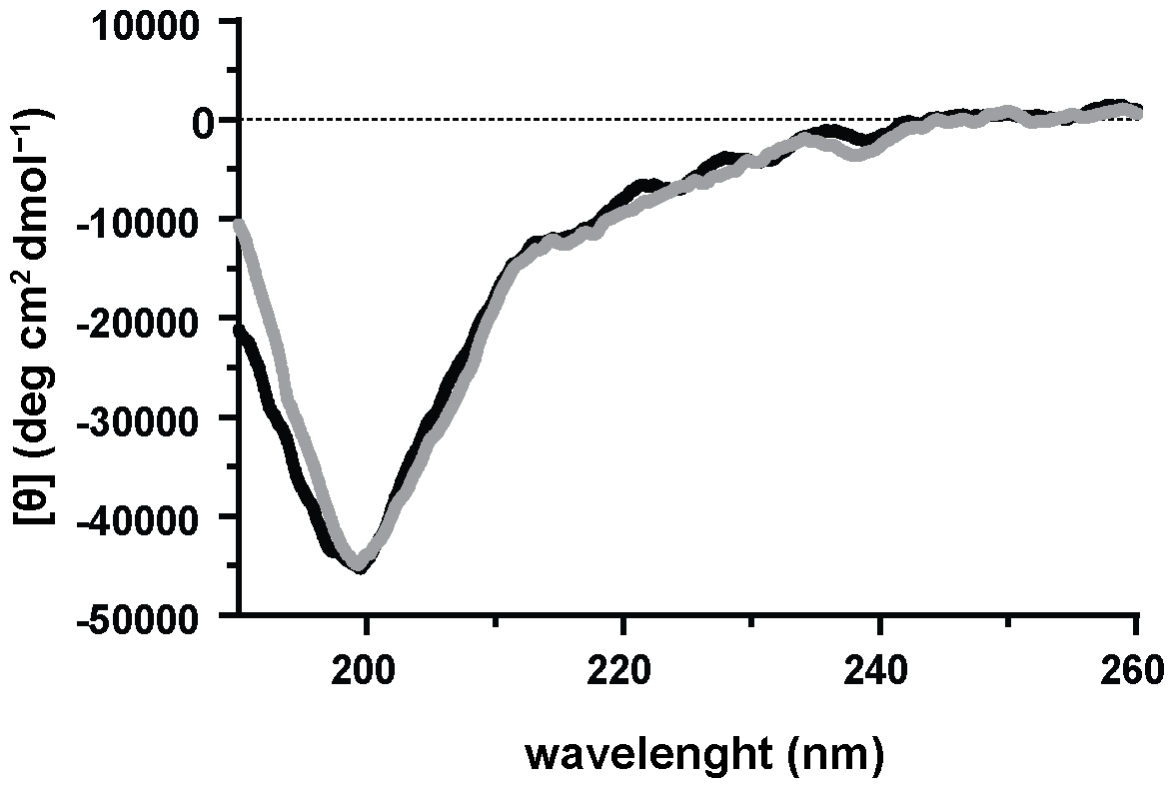
Madanins are intrinsically disordered in solution. Circular dichroism spectra of purified recombinant madanin-1 (black) and madanin-2 (gray) were recorded in the far-UV region (190–260 nm), revealing the unstructured nature of the polypeptides.

Madanin-1 and madanin-2 were expressed in *E. coli* as recombinant proteins and purified by a two-step protocol: affinity chromatography on chitin-agarose followed by size-exclusion chromatography. The integrity of the purified recombinant proteins was verified by mass spectrometry and N-terminal sequencing. The MALDI-MS spectra of purified recombinant madanin-1 and madanin-2 revealed major peaks at 6766.5 Da and 7122.2 Da, respectively, in good agreement with the predicted molecular masses of the full-length forms of the proteins: 6770.2 Da for madanin-1 and 7122.5 Da for madanin-2. Furthermore, both molecules displayed an intact N-terminus, as indicated by the sequence (Tyr-Pro-Glu-Arg) obtained after four cycles of Edman degradation. Finally, circular dichroism spectra of recombinant madanin-1 and madanin-2 are characteristic of “random-coils” suggesting that both molecules are intrinsically disordered in solution (Figure 1).

### Madanins are competitive thrombin inhibitors

**Figure 2:**
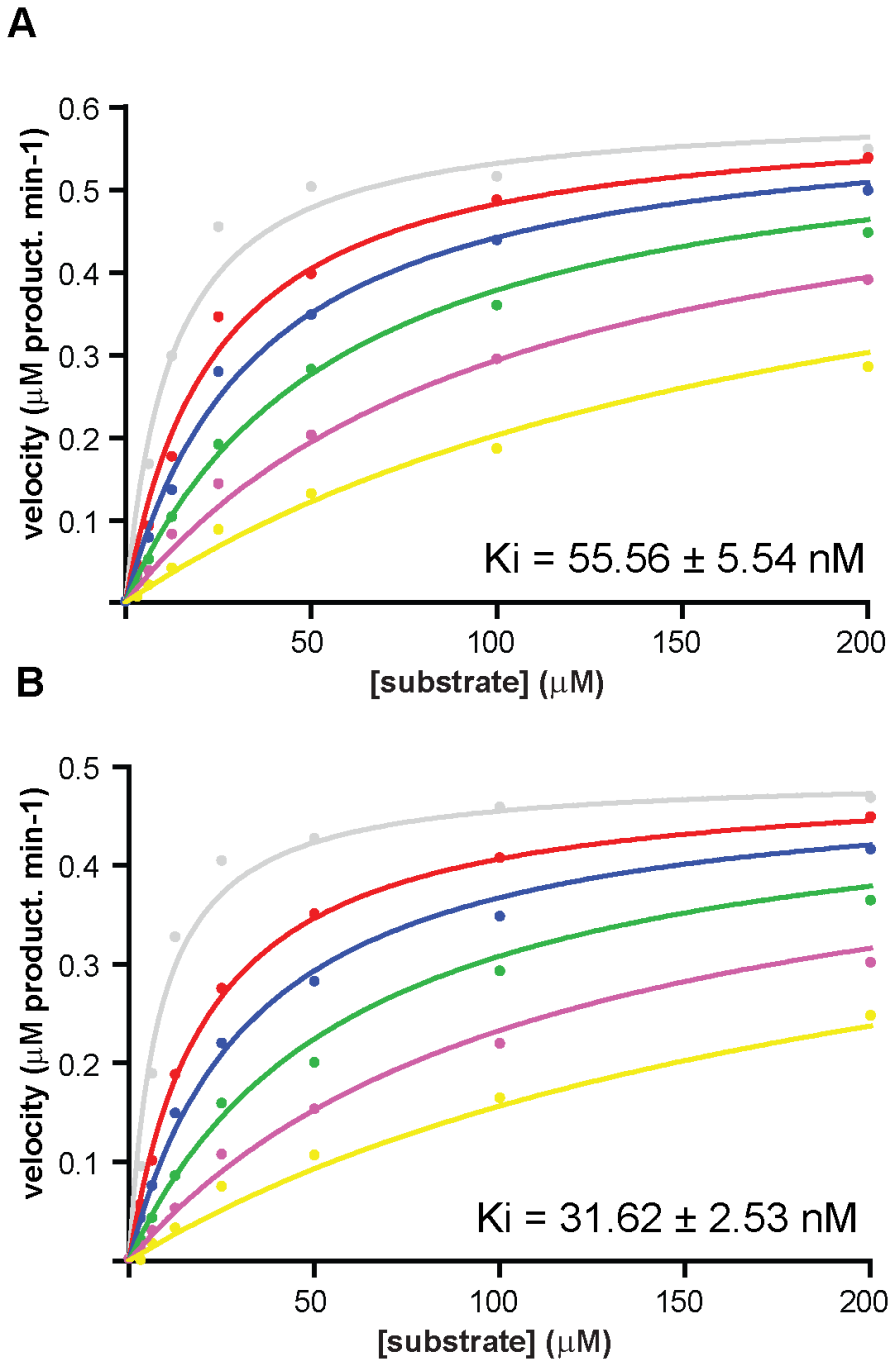
Madanins are competitive inhibitors of α-thrombin. α-Thrombin (1 nM) hydrolysis of the small chromogenic substrate Tos-Gly-Pro-Arg-p-nitroanilide (0–200 µM) in the absence or in the presence of increasing concentrations (0 nM grey, 50 nM red, 100 nM blue, 200 nM green, 400 nM violet, 800 nM yellow) of madanin-1 (A) or madanin-2 (B) shows binding competition for the active site. Kinetic parameters of α-thrombin inhibition (*K*_i_ values ± SEM) given are representative of three independent experiments. Data was fitted to the competitive inhibition model with GraphPad Prism 5 (GraphPad Software).

In striking contrast to a previous report [7], we found that under low ionic strength conditions (50 mM NaCl), madanin-1 and madanin-2 compete with a small synthetic chromogenic substrate (Tos-Gly-Pro-Arg-p-nitroanilide) for binding to the active site of thrombin, inhibiting the enzyme in a dose-dependent manner (Figure 2). In this experimental setting, madanin-1 and madanin-2 display inhibition constants (*K*_i_) for thrombin of 55.56 ± 5.54 nM and 31.62 ± 2.53 nM, respectively. Furthermore, both recombinant madanins display anticoagulant activity *in vitro*, as indicated by the dose-dependent prolongation of thrombin time (TT). Madanin-1 and madanin-2 doubled TT at a concentration of approximately 5 µM (Table 2), a result that is in agreement with the data previously reported for recombinant madanins containing an additional N-terminal methionine residue [7].

**Table 2:** Madanin-1 and madanin-2 inhibit plasma clotting by prolongation of thrombin time.

	Madanin-1		Madanin-2	
Concentration ( µM)	Time (s)	Prolongation (fold)	Time (s)	Prolongation (fold)
0	17.5 ± 0.5	1.00	17.5 ± 0.5	1.00
5	36.0 ± 0.5	2.00	33.0 ± 0.5	1.83
10	52.5 ± 0.5	2.89	47.5 ± 1.0	2.61

### Madanins are processed by thrombin upon complexation

**Figure 3:**
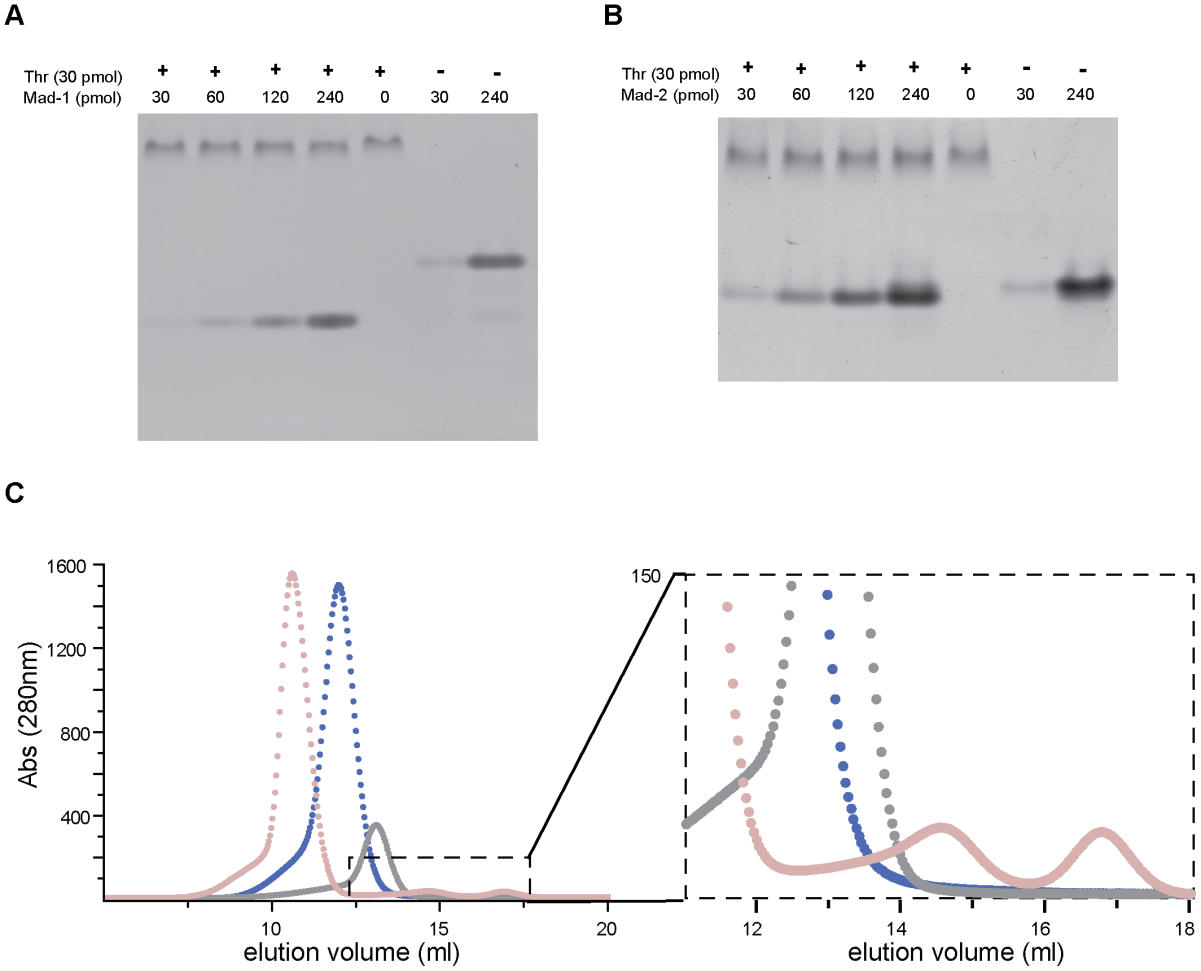
Formation of thrombin·madanin complexes *in vitro*. (A) Thrombin·madanin-1 and (B) thrombin·madanin-2 complex formation was assessed using an electrophoretic mobility shift assay. Samples were separated in non-denaturing 10% polyacrylamide gels and stained with PageBlue (Fermentas). The amounts of thrombin (Thr), madanin-1 (Mad-1) and madanin-2 (Mad-2) are indicated. (C) Thrombin·madanin-1 complex formation was assessed by size exclusion chromatography. Human α-thrombin was mixed with a 10% molar excess of purified recombinant madanin-1. The complex was separated from excess inhibitor by size exclusion chromatography on a Superdex75 HR 10/30 column (pink). For comparison, thrombin (blue) and recombinant madanin-1 (grey) were individually applied to the same column. The magnification of the chromatogram region enclosed in a dashed rectangle (right) shows the formation of low-molecular weight products after incubation of excess madanin-1 with the proteinase.

**Figure 4:**
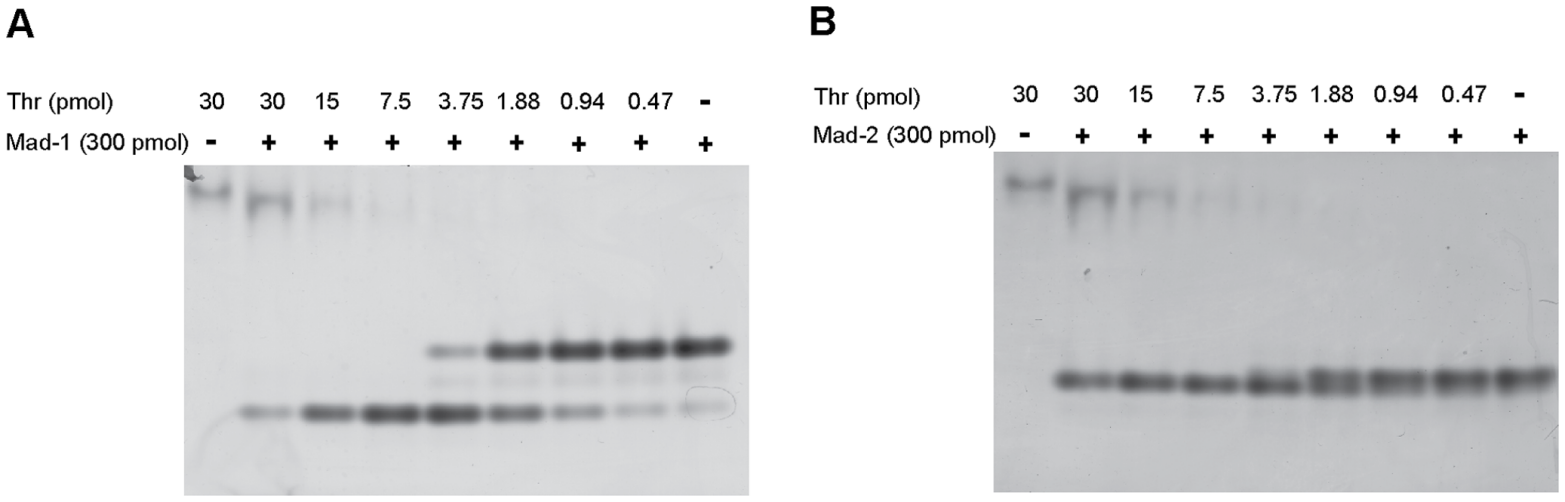
Madanins are modified by α-thrombin. (A) Madanin-1 or (B) madanin-2 was treated with the indicated amounts of thrombin for 30 min at 37°C. Samples were separated in a non-denaturing 10% (w/v) polyacrylamide gel. Notice the appearance of additional species migrating faster than the intact inhibitors in the presence of thrombin, in a dose-dependent manner.

Complexes between human α-thrombin and madanin-1 or madanin-2 were prepared *in vitro* and analyzed on an electrophoretic mobility shift assay. A fixed amount of thrombin (30 pmol) was incubated with increasing amounts of purified inhibitor and separated in a non-denaturing gel. When thrombin and madanin-1 or madanin-2 were mixed in equimolar amounts (Figure 3A and 3B, lane 1) formation of a single species with a slightly different migration profile from thrombin alone (Figure 3A and 3B, lane 5) was observed, suggesting the formation of a 1∶1 thrombin·madanin complex. However, bands migrating faster than free madanin were observed when a molar excess of the inhibitor was used (Figure 3A and 3B, lanes 2–4), indicating possible processing of madanins by thrombin. Furthermore, the fast-migrating species observed in non-denaturing gel electrophoresis are protease dose-dependent, as demonstrated by electrophoretic mobility shift assays performed with a fixed amount of madanin-1 or madanin-2 (300 pmol) and a range of thrombin concentrations (Figure 4). The mobility shift between intact madanin and the thrombin-cleaved inhibitor is significantly larger for madanin-1 than for madanin-2, despite the similar mass, charge, and isoelectric point of both inhibitors (Figure 4). However, it is conceivable that the differences in the amino acid composition of madanin-1 and madanin-2, albeit small, may originate cleavage fragments with distinct isoelectric points and hence different behavior in the electrophoretic mobility shift assay.

Size-exclusion chromatography analysis of a mixture of α-thrombin with 10% molar excess of madanin-1 (Figure 3C, pink) not only shows the formation of a species of higher molecular weight than free thrombin (Figure 3C, blue), apparently corresponding to the binary complex, but also the appearance of products (Figure 3C inset, pink) of lower molecular weight than free madanin-1 (Figure 3C, grey). Altogether, these results suggest cleavage of madanins by thrombin at least at one site.

### Thrombin cleaves MEROPS family I53 inhibitors

**Figure 5:**
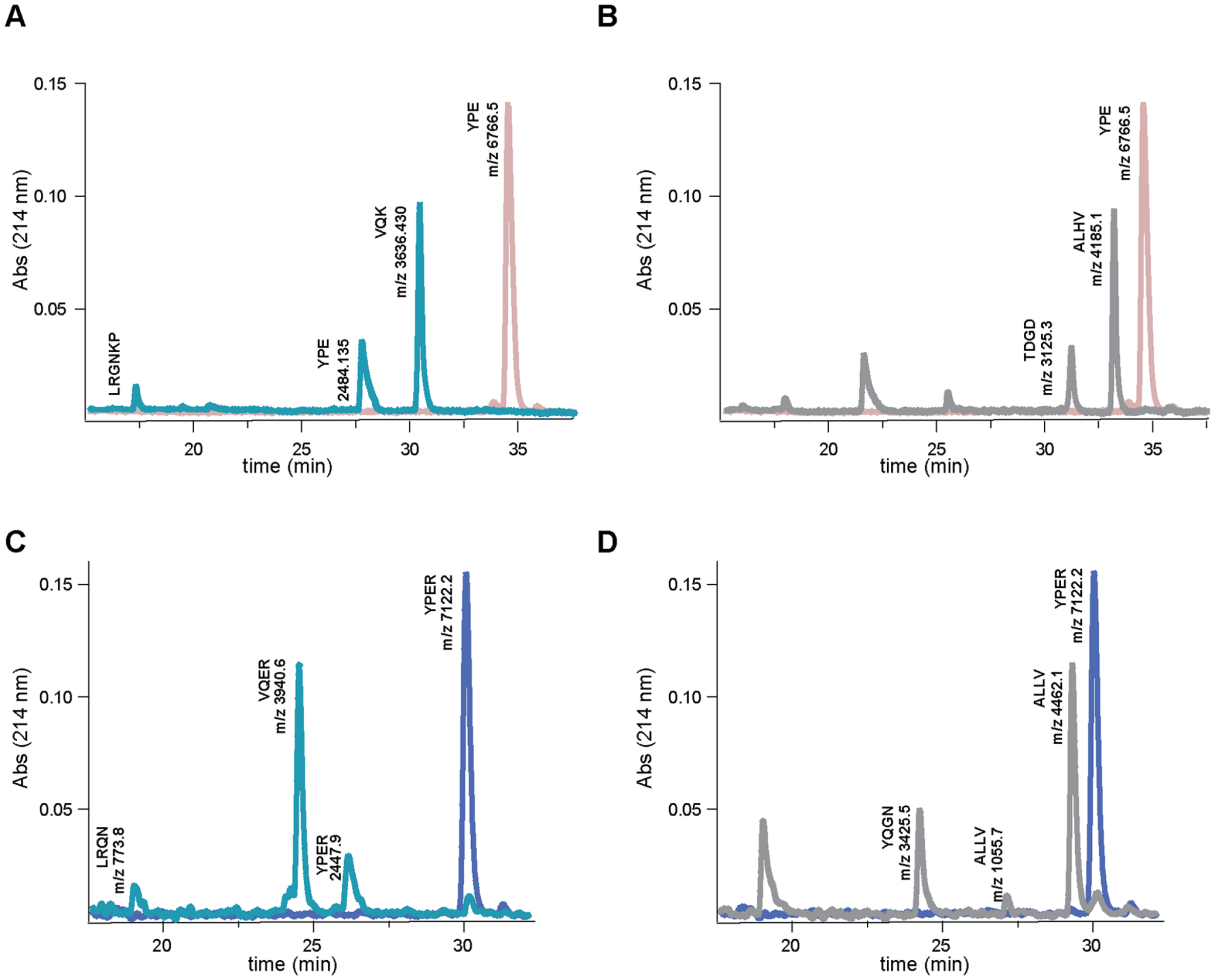
Madanins are cleaved at specific sites by both α-thrombin and factor Xa. Madanins were incubated in the absence and in the presence of human α-thrombin or bovine factor Xa at 37 °C for 2 hours. The products were separated by reverse-phase HPLC and analysed by MS and N-terminal sequencing. (A and B) Reverse-phase chromatograms of intact (pink) or cleaved (thrombin, cyan; factor Xa, grey) madanin-1 (C and D). Reverse-phase chromatograms of intact (blue) or cleaved (thrombin, cyan; factor Xa, grey) madanin-2. For each peak the experimental molecular mass obtained by MS (m/z) and the N-terminal sequences results are indicated.

Proteases preferentially cleave substrates within extended loop regions and the cleavage sites are dictated by the specificity of the enzyme [30]. N-terminal sequencing and mass spectrometry analysis of the madanin-1 and madanin-2 hydrolysis products, following separation by reverse-phase HPLC, allowed the identification of the thrombin cleavage sites. Intact madanin-1 and madanin-2 elute from the reverse-phase column as single peaks, having the expected molecular masses in MS analysis (Figure 5, Table 3). Upon incubation with thrombin, peaks with shorter retention times could be detected, corresponding to inhibitor cleavage products (Figure 5A, 5C). For madanin-1, three fragments could be identified: fragment 1, corresponding to madanin-1 residues 1-21; fragment 2, corresponding to residues 22–54; and fragment 3, corresponding to the C-terminal peptide of the inhibitor (residues 55–60; Figure 5A, Table 3). For madanin-2, the three major peaks correspond to residues 1–21, 22–55, and 56–61, respectively (Figure 5C, Table 3).

**Table 3:** Thrombin and factor Xa cleave madanin-1 and madanin-2.

Inhibitor	Protease	Fragment	N-terminal sequence	Molecular mass (Da)		Residue range
				Experimental	Theoretical	
Madanin-1	None	–	Tyr-Pro-Glu-Arg	6766.5	6770.2	1–60
Madanin-1	α-Thrombin	1	Tyr-Pro-Glu	2484.1	2484.6	1–21
Madanin-1	α-Thrombin	2	Val-Gln-Lys	3636.4	3637.7	22–54
Madanin-1	α-Thrombin	3	Leu-Arg-Gly-Asn-Lys-Pro	–	683.8	55–60
Madanin-1	Factor Xa	1	Thr-Asp-Gly-Asp	3125.3	3126.1	26–54
Madanin-1	Factor Xa	2	Ala-Leu-Lys-Val	4185.1	4186.4	17–54
Madanin-2	None	–	Tyr-Pro-Glu-Arg	7122.2	7122.5	1–61
Madanin-2	α-Thrombin	1	Tyr-Pro-Glu-Arg	2447.9	2446.7	1–21
Madanin-2	α-Thrombin	2	Val-Gln-Glu-Arg	3940.6	3939.0	22–55
Madanin-2	α-Thrombin	3	Leu-Arg-Asn-Gln	773.8	772.8	56–61
Madanin-2	Factor Xa	1	Tyr-Gln-Gly-Asn	3425.5	3426.4	26–55
Madanin-2	Factor Xa	2	Ala-Leu-Leu-Val	1055.7	1055.2	17–25
Madanin-2	Factor Xa	3	Ala-Leu-Leu-Val	4462.1	4463.7	17–55

**Figure 6:**
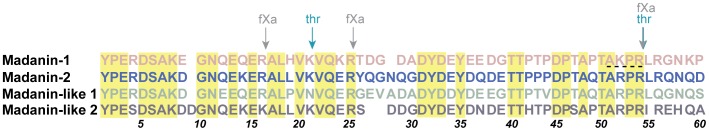
Sequence alignment of MEROPS family I53 inhibitors from the bush tick *Haemaphysalis longicornis*. Alignment of the amino acid sequences of the mature forms of madanin-1 (UniProt entry Q86FP9_HAELO) and madanin-2 (Q86FP8_HAELO), and of the putative mature madanin-like protein 1 (Q4R1A5_HAELO) and madanin-like protein 2 (Q4R1A2_HAELO). Strictly conserved residues are highlighted in yellow. The numbering for mature madanin-1 is given below the alignment. Thrombin (teal) and factor Xa (grey) cleavage sites are indicated. The dashed line highlights the four residues of madanin-1 modeled in the experimental three-dimensional structure of the thrombin·madanin complex.

As expected, in all cases a basic amino acid residue could be found at position P1, preceded by a small hydrophobic side chain in P2, in good agreement with thrombin's known substrate specificity [31] (Figure 6). Both thrombin cleavage sites are conserved in madanin-like 2, while in madanin-like 1 the residue topologically equivalent to Lys21 of madanin-1 has been replaced by an asparagine (Figure 6).

The upstream cleavage site for both madanin-1 (Leu-His-Val-Lys | Val-Gln-Lys-Arg) and madanin-2 (Leu-Leu-Val-Lys | Val-Gln-Glu-Arg) resembles the motif found in the physiological substrate protease-activated receptor (PAR)-3 (Leu-Pro-Ile-Lys | Thr-Phe-Arg-Gly) bearing an aliphatic P4 residue (leucine) and a lysine residue at the P1 position. However, thrombin has a strong preference for arginine over lysine at this critical position [31], and the overwhelming majority of thrombin cleavage sites in natural substrates possess a P1 arginine residue. Therefore, positioning of madanin's Lys21-Val22 peptide bond into the active site of the proteinase in a productive conformation might depend on secondary interactions with one of thrombin's exosites. Indeed, the structure of the thrombin·variegin complex [16] suggests that the hirudin-like motif Asp31-Asp38 in madanin-1 might engage in important contacts with thrombin exosite I residues, similar to the acidic peptides found in variegin [16] and in hirudin [32]–[34], but also in physiological thrombin substrates such as factor V [35] and PAR1 [36].

Furthermore, the downstream cleavage site for both madanin-1 (Ala-Lys-Pro-Arg | Leu-Arg-Gly-Asn) and madanin-2 (Ala-LysArg-Pro-Arg | Leu-Arg-Gln-Asn) resembles the motif found in the physiological substrates protein C (Val-Asp-Pro-Arg | Leu-Ile-Asp-Gly), coagulation factors XI (Ile-Lys-Pro-Arg | Ile-Val-Gly-Gly) and XIII (Val-Val-Pro-Arg | Gly-Val-Asn-Pro), and insulin-like growth factor (Met-Val-Pro-Arg | Ala-Val-Tyr-Leu) [37]. The latter confirms the strong preference of thrombin for substrates with an arginine in position P1 and proline in position P2. In this case, exosite-mediated interactions are not required to position the P1 (Arg54) – P1' (Leu55) scissile peptide bond into the protease active site cleft in an appropriate conformation for cleavage, as experimentally shown below.

### Madanins are also processed by coagulation factor Xa

The proteolytic factor preceding thrombin in the coagulation cascade, factor Xa, has a similar preference for substrates with basic residues at position P1 [38]-[41]. Therefore, the ability of factor Xa to hydrolyze madanin-1 and madanin-2 was assessed, following an approach similar to that described above for α-thrombin. Purification of cleavage products by RP-HPLC, followed by N-terminal sequencing and MS analysis allowed the identification of three factor Xa cleavage sites on each of the inhibitors: Glu-Gln-Glu-Arg | Ala-Leu-His-Val, Val-Gln-Lys-Arg | Thr-Asp-Gly-Asp, and Ala-Lys-Pro-Arg | Leu-Arg-Gly-Asn for madanin-1 and Glu-Lys-Glu-Arg | Ala-Leu-Leu-Val, Val-Gln-Glu-Arg | Tyr-Gln-Gly-Asn, and Ala-Arg-Pro-Arg | Leu-Arg-Gln-Asn for madanin-2 (Figure 6). For madanin-1, two fragments could be identified: fragment 1, corresponding to residues 26–54, and fragment 2, spanning residues 17–54 (Figure 5B, Table 3). For madanin-2, three fragments could be characterized: fragment 1, corresponding to residues 26–55; fragment 2, corresponding to residues 17–25; and fragment 3, corresponding to residues 17–55 (Figure 5D, Table 3).

Thus, factor Xa was found to cleave both madanin-1 and madanin-2 exclusively after arginine residues. The Glu-Gln-Glu-Arg | Ala-Leu-His-Val and Val-Gln-Lys-Arg | Thr-Asp-Gly-Asp cleavage sites in madanin-1 and the topologically equivalent sites in madanin-2 (Figure 6) were recognized as substrate motifs by factor Xa but not by thrombin. This is partially explained by the presence of bulky polar amino acids at position P2 that prevent cleavage by thrombin, with a known strong preference for proline or aliphatic residues at this position [37]. Furthermore, the C-terminal Ala-(Lys/Arg)-Pro-Arg | Leu-Arg-(Gly/Gln)-Asn motif was hydrolyzed by both coagulation factors. The factor Xa cleavage sites are also conserved in madanin-like 1 and madanin-like 2 proteins, with the exception of the most N-terminal site, where the putative P1 residue has been replaced by lysine in madanin-like 2 (Figure 6).

### Thrombin-cleaved madanins are devoid of anti-clotting activity

The madanin-1 and madanin-2 fragments resulting from thrombin processing were purified and assayed for anticoagulant activity. Full-length inhibitors subjected to reverse-phase chromatography and lyophilization (Table 4) prolonged thrombin time similarly to unprocessed recombinant proteins (Table 2). Unlike the full-length polypeptides (Table 4), the madanin cleavage fragments did not significantly prolong thrombin time, suggesting that the hydrolysis products are unable to compete with thrombin's natural substrate, fibrinogen, for binding to the enzyme. In agreement, the fragments only marginally affected the amidolytic activity of thrombin against a chromogenic substrate *in vitro* when used at 1000-fold molar excess (Table 5).

**Table 4:** Thrombin-generated fragments of madanin-1 and madanin-2 do not inhibit plasma clotting.

	Madanin-1		Madanin-2	
Concentration ( µM)	Time (s)	Prolongation (fold)	Time (s)	Prolongation (fold)
0	17	1.00	17	1.00
10	50	2.94	44	2.59
	Fragment 55–60		Fragment 56–61	
10	17	1.00	17	1.00
	Fragment 1–21		Fragment 1–21	
10	17	1.00	18	1.06
	Fragment 22–54		Fragment 22–55	
10	20	1.18	18	1.06

**Table 5:** Madanin-1 and madanin-2 hydrolysis products do not inhibit thrombin amidolytic activity.

	Thrombin inhibition (%)
No inhibitor	0.00
Madanin-1 (1 µM)	43.75 ± 0.78
Madanin-2 (1 µM)	47.17 ± 1.01
Madanin-1 fragment 1–21 (1 µM)	4.60 ± 0.33
Madanin-1 fragment 22–54 (1 µM)	5.77 ± 0.42
Madanin-2 fragment 1–21 (1 µM)	5.83 ± 0.53
Madanin-2 fragment 22–55 (1 µM)	6.11 ± 1.48

### Crystal structure of thrombin in complex with a post-cleavage madanin-1 fragment

**Figure 7:**
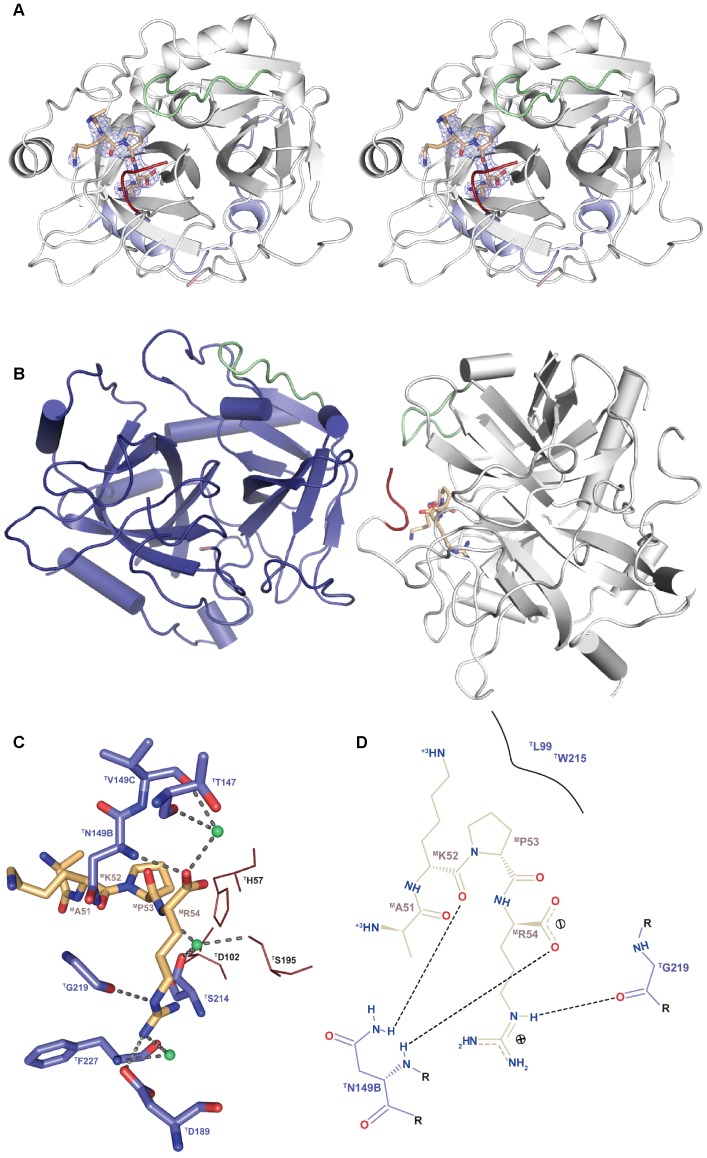
A product of madanin-1 hydrolysis remains bound to the active site of α-thrombin. (A) Stereographic representation of the human α-thrombin·madanin-1 complex. The thrombin molecule is represented as a grey cartoon and shown in the standard orientation for serine proteinases, i.e. substrates run from “left” to “right”. The madanin-1 fragment is depicted in sticks representation with nitrogen atoms in blue, oxygen in red and carbon in yellow. The 60-loop and the 149-loop of thrombin are colored in green and red, respectively. The unbiased Fo-Fc electron density map (2σ cut-off) for the madanin-1 tetrapeptide (Ala51-Arg54) is displayed as a blue mesh. (B) The asymmetric unit contains two thrombin molecules, represented in gray and blue cartoons with alpha helices and beta strands represented as cylinders and arrows, respectively. The 60- and 149-loops of thrombin are colored as in (A). A madanin-1 tetrapeptide bound to the active site of one of the thrombin molecules and packed against the 149-loop of the neighboring protease moiety is depicted as in (A). (C) Close-up view of the interactions between the madanin-1 fragment (colored as in (A)) and the active site residues of thrombin (selected residues represented as sticks color-coded as madanin-1 except for carbon atoms, colored teal blue). The active site residues ^T^H57, ^T^D102 and ^T^S195 of thrombin are colored red. Water molecules are represented as green spheres. (D) Schematic representation of the main interactions established between human thrombin and the madanin-1 fragment. Hydrogen bonds are represented as dotted black lines. Figure prepared with PyMOL (http://www.pymol.org) and PoseView [48].

The three-dimensional structure of human α-thrombin in complex with a cleavage fragment of madanin-1 was determined by X-ray crystallography at a resolution of 2.6 Å. The crystals belong to the orthorhombic space group P2_1_2_1_2_1_ and contain two proteinase moieties and a madanin-1 fragment in the asymmetric unit. Each of the two proteinase moieties adopts a structure very similar to that of active ligand-free human thrombin ([27], PDB entry 3U69), with a r.m.s.d. of 0.33–0.43 Å (249–251 aligned Cα atoms) for thrombin chains AB and LH, respectively, with most differences being attributable to crystal packing effects. One of the thrombin molecules (chains A and B) is ligand-free, while at the active site of the other proteinase molecule (chains L and H) there is interpretable electron density for madanin-1 residues Ala51 to Arg54 (Figure 7A). The ligand-free thrombin molecule packs against the cleaved autolysis loop of the other proteinase molecule, which is therefore unusually ordered, with the exception of Lys149E that is not defined in the electron density maps. Residues of the 149-loop interact both with the bound madanin-1 fragment (see below) and the other thrombin molecule in the asymmetric unit, occluding the enzyme's active site and impairing the release of this madanin-1 cleavage product (Figure 7B).

The strict substrate specificity of thrombin results from the combination of the narrow, canyon-like structure of its active site cleft with the unique insertion loops that restrict the access to the catalytic center (60-loop, Tyr60A to Ile60I and 149-loop, Thr149A to Ala149E). The ability of thrombin to cleave its substrates is dependent of the attack of the hydroxyl oxygen of the catalytic Ser195 to the carbonyl carbon of the substrate P1 residue. Due to the acidic nature of thrombin's S1 specificity pocket, conferred by the presence of Asp189 at its bottom, arginine and lysine are thrombin's preferred P1 residues. Accordingly, in the current structure of the madanin-1·thrombin complex, the side chain of madanin-1 residue ^M^Arg54 occupies thrombin's S1 site, with a placement reminiscent of other natural substrates and inhibitors [15], [36], [42]. The guanidinium group of ^M^Arg54 establishes several polar contacts with ^T^Asp189 OD1 and the carbonyl oxygen atoms of ^T^Gly219, ^T^Trp215, and ^T^Phe227, which are strengthened by hydrophobic contacts between the ^M^Arg54 side chain and the ^T^Cys191-^T^Cys220 disulfide bond. Further, well-ordered water molecules connect the main chain nitrogen of ^M^Arg54 and both the hydroxyl group of the catalytic ^T^Ser195 residue and the carbonyl oxygen of ^T^Ser214. Finally, the carbonyl oxygen of ^M^Arg54 establishes a direct hydrogen bond to ^T^Asn149B N and a water-mediated interaction with ^T^Val149C O. The increased distance (5.4 Å) between the carbonyl carbon of the P1 residue and the hydroxyl oxygen of the catalytic ^T^Ser195 is in agreement with the post-hydrolysis status of the bound madanin-1 moiety (Figure 7C, 7D).

Natural thrombin substrates often possess a Pro residue at position P2 [31]. In madanin-1, the favorable ^M^Pro53 at this position makes Van der Waals interactions with the 60-loop residues ^T^Tyr60A and ^T^Trp60D, as well as with ^T^Leu99 and the catalytic ^T^His57, contacts that are common to several thrombin substrates [36], [42]–[46]. Additionally, there is a direct interaction between ^M^Lys52 O and ^T^Asn149B ND2, as well as Van der Waals contacts with the side chain of ^T^Ile174, while the upstream ^M^Ala51 makes only minor contacts with the proteinase, being therefore rather disordered in the electron density (Figure 7C, 7D).

The bound madanin-1 fragment in this crystal structure fits well the substrate preferences of thrombin, particularly at positions P1 and P2, explaining the enzyme's preference for cleavage of the ^M^Arg54-^M^Leu55 peptide bond. The non-canonical position of the side chain of ^M^Arg54 and the increased distance between the peptide C-terminus and the catalytic residues is compatible with the post-cleavage nature of the complex.

### A superfamily of cleavable anticoagulants?

As for other standard inhibitors of serine proteinases, i.e. those following the Laskowski mechanism of inhibition [47], madanins bind to thrombin similar to a substrate, as clearly illustrated by the three-dimensional structure of the post-cleavage complex here reported. However, unlike standard inhibitors, the cysteine-less madanins lack the frequently observed covalent stabilization of the reactive loop, while the absence of a compact core impacts their ability to establish favorable secondary interactions with the proteinase. Consequently, madanins are quickly and extensively processed by thrombin, and the resulting fragments are devoid of inhibitory activity. Therefore, madanins seemingly exert their inhibitory role by outcompeting the enzyme's natural substrates. This is in striking contrast to the other structurally characterized cysteine-less thrombin inhibitors, variegin [16] and anophelin [15]. The latter eludes proteolytic processing by thrombin by employing a unique reverse-binding mode and by specifically disrupting the enzyme's active site [15]. As for variegin, despite binding to and being processed by thrombin in a manner similar to substrates, the C-terminal cleavage product displays significant affinity for the proteinase and anticoagulant activity [16].

Despite the lack of sequence similarity between madanins and variegin, the mechanism by which they oppose their proteolytic targets in the coagulation cascade shares evident resemblances. Together with the conserved features (e.g. a hirudin-like acidic stretch - Asp31-Asp38 in madanin-1) in their primary structures, this might justify grouping these inhibitors in a wider superfamily, where they might eventually be joined by the chemically similar chimadanin (MEROPS family I72; [12]), whose mechanism of action remains to be disclosed.
